# Perceived Effects of Socio-Economics and Social Media Variables on Body Mass Index in Saudi Young Adults

**DOI:** 10.7759/cureus.30349

**Published:** 2022-10-16

**Authors:** Suzan H Tami

**Affiliations:** 1 Department of Clinical Nutrition, College of Applied Medical Sciences, King Faisal University, Hofuf, SAU

**Keywords:** health promotion, nutrition information, young adults, social media, behavioral nutrition

## Abstract

This study aimed to measure the extent to which Saudi young adults used social media as a source for nutrition information and assess the perception of those networks on their dietary behaviors. The study also investigated the association between participants' socio-economic variables, social media variables, and body mass index. A cross-sectional survey was used and distributed via social media. This study included 228 participants (20-24 years old) who lived in AL-Ahssa Province. Over 50% of the participants were single females with bachelor's degrees. Among the participants, 70% were interested in increasing their nutrition knowledge, and 95% agreed that they obtained their nutrition information through social media. The participants surfed social media for at least one hour per day. Over 70% responded that exposure to social media had influenced their dietary behaviors (adopting new positive dietary behaviors, including selecting healthy foods and reading nutrition labels). Instagram was the most used application of all social media networks, with over 50% of the participants following 2-4 Instagram accounts related to nutrition. A majority of participants (78%) preferred obtaining nutrition information through visual methods, such as videos (49.1%) and infographic images (28.9%). Challenges to using nutrition information on social media were unproven or misleading topics and provided by non-nutrition experts. Social media may therefore be an effective tool to increase nutrition awareness and assist in disseminating nutrition intervention programs by nutrition educators and experts.

## Introduction

The rapid and prolific development of information technology and social media has contributed to the communication between individuals [1,2]. Notably, young adults rely on social media networks for communication in different forms (texts, videos, podcasts, and infographics). In Saudi Arabia, the 2019 General Authority for Statistics report results indicated that 98.43% of young adults (ages 15-34) used social media networks [3]. Compared to other Saudi regions, the Western region (including Al-Ahssa Province) had the highest rate (95.43%) of internet users [4]. The highest internet user prevalence (96.56%) was among 20-24 years old.

According to the Saudi Health Information Survey that collected data from 2,382 youths (aged 15 to 24 years old), 45.9 % of males and 48.4 % of females had normal body mass index (BMI) [5]. Male youth were more likely than female youth to smoke cigarettes or shisha. The prevalence of daily consumption of at least five servings of fruits and vegetables was 6.6 %. The prevalence of no or insufficient physical activity was 41.8 % in males and 75.6 % in females (P < 0.001). About 40 % of males and 25 % of females had abnormal blood pressure. The survey findings concluded that mean BMI, insufficient physical activity, current smoking, and high blood pressure were higher in the 20-24-year-old population than in younger ages. Moradi-Lakeh et al. concluded that if these behaviors are not reversed during this critical age period, the disease burden will increase in Saudi Arabia in the coming years, suggesting more attention and resources for intervention [5].

There is an excellent opportunity for young adults to utilize social media networks to obtain different nutritional information [6]. However, these networks must be used cautiously to avoid misleading information and minimize potential adverse effects. In addition, the reliability of nutrition information from social media network might be lacking, especially since some nutritional messages are provided by non-experts (unreliable influencers from outside the nutrition field) [7,8]. In particular, these influencers would compete to attract young adults' interest, using various interactive ways to deliver their messages through social media networks. Moreover, high-frequency social media use during the day might be associated with physical inactivity [9,10]. 

Social media networks allow public health professionals to disseminate nutrition information to young adults. Using social media networks for disseminating nutrition information to young adults is an art. It requires a nutritionist to partner with other communications, media, and marketing experts to understand how to interact with young adults [2]. It also needs a careful selection of information, resources, and strategies to maintain ethical and professional standards for all activities being offered. Previous research assessed sources from which young adults in different societies (such as Australian, Greek, and Ghanaian) obtain nutrition information and found that the internet, specifically social media networks, were a trusted resource of health and nutrition information for this age group [11-13].

However, no research has addressed whether Saudi young adults use and rely on social media networks to obtain nutrition information. Such data is needed to develop nutrition education programs that promote a lifespan of healthier nutrition behaviors and better health. Thus, the current study aimed to assess the use of social media networks as a source of nutrition information in Saudi young adults in Al-Ahssa Province. In addition, the study examined the association between socio-economics and social media variables and participants' BMI.

## Materials and methods

Study design and participants

Using a convenience sampling method, the data of this cross-sectional study were collected through a self-completed online survey created using Google Forms. The study survey link was distributed via social media networks (Twitter- Instagram- Snapchat) and targeted Saudi young adults (20-24 years old) from Al-Ahssa Province who were not nutrition majors to avoid bias. Also, several Saudi public figures (nutrition and public health researchers and social media influencers) with a high number of Saudi Arabian followers were asked to share the study survey link on their social media channels. Respondents to the study survey were asked to provide consent before participating and were able to exit the survey at any point. After reviewing the dataset to identify duplicate or incomplete data, the final dataset included 228 participants

Measures

The survey questions collected demographic data, including sex, age, weight, height, occupation, marital status, education level, origin city (Al-Hufof, Al Mubarraz, or Ahsa village), and household income (income of less than SR 6,000 referred to a low-income household, and over SR 19,000 referred to a high-income household). The survey also included questions about the participants' interests and the resources used to increase their nutrition awareness. In addition, the participants were asked about the amount of time they spent surfing different social media networks (Twitter- Instagram- Snapchat) and the number of accounts providing nutrition information they followed.

Furthermore, the participants were asked about which social media networks they depended on the most in increasing their nutritional information and how these networks affected their dietary behaviors. Moreover, the participants were asked about nutrition-related topics they were interested in and in which forms (text, infographic images, video, podcast) they preferred to obtain nutritional information. The participants were allowed to choose multiple responses to the questions regarding nutrition-related topics they were interested in and the forms they preferred to obtain nutritional information. The study protocol was approved by King Faisal University's Research Ethics Committee (KFU-REC/2020-05-16).

The nutrition and social media questions were devised for this study. However, prior to the current study, test-retest reliability and internal consistency of the survey questions were assessed (data not shown) with 38 respondents, with at least a one-week interval (7-14 days) between the two administrations. The 38 respondents (age mean= 22.31±1.37 years old) were not major in nutrition and were recruited through a snowball-sampling method. Pearson's correlation of the survey questions was 0.74, and the estimated internal consistency reliabilities (Cronbach's alpha) of the survey questions increased from 0.74 for the first administration to 0.93 for the second administration (data not shown). High correlations and estimated reliabilities suggested acceptable test-retest reliability and internal consistency of the questions.

Data analysis

The demographic characteristics of the participants were analyzed, including descriptive statistics such as proportions, frequencies, percentages, means, and standard deviations. Frequency distributions were used to display the numbers and percentages of the participants' interest and the resources used in increasing their nutrition awareness, participants' usage amounts of time spent surfing different social media networks, the number of followed social media accounts providing nutrition information, perceptions on how social media networks affected their dietary behaviors, nutrition-related topics forms that participants preferred, and food delivery applications usage. The study data were analyzed using the Statistical Package for Social Sciences software (SPSS, v. 27, 2020).

Using participants' self-reported height and weight, each participant's BMI was calculated (with the formula of BMI = Kg/m^2^, where kg is a person's weight in kilograms, and m^2^ is their height in meters squared) [14]. A BMI of less than 18.5 is considered underweight, while a normal/healthy weight BMI is 18.5 to 24.9. A BMI of 25.0 to 29.9 is overweight, and a BMI of 30 or more is considered obese. Using R software, the Tobit model (censored normal regression), designed to estimate linear relationships between variables when there is either left- or right-censoring in the dependent variable, was used to examine the effect of socio-economic variables and social media variables on participants' BMI. The Tobit model is expressed as below [15]:

(1) 𝒚𝒊 ∗ = 𝒙𝒊 ′𝜷 + 𝜺𝒊

(2) 𝒚𝒊 = { 𝒂 𝒊𝒇 𝒚𝒊 ∗ ≤ 𝒂 𝒚𝒊 ∗ 𝒊𝒇 𝒂 < 𝒚𝒊 ∗ < 𝒃 𝒃 𝒊𝒇 𝒚𝒊 ∗ ≥ 𝒃

In the model, 𝒚𝒊 is the BMI, 𝒙𝒊 ′ are explanatory variables, 𝜺𝒊 the error term, 𝒂 is the lower limit of the BMI score, and 𝒃 is the upper limit of the BMI score.

## Results

Table 1 shows the characteristics of the young Saudi adult participating in this study. Among 228 study participants, over 50% of the participants were female (n = 127), single (n = 129), university student (n =123), from Hofuf and Al-Mubarraz cities (n = 155), having a bachelor’s degree (n = 144), and had a monthly household income of less than SR 9,999 (n = 128). In addition, the mean age of the participants was 22 (SD = 1.5), and the mean BMI was 25.4 (SD = 5.6).

**Table 1:** Characteristics of the Saudi young adult participants (n = 228)

Characteristic	N (%)
Sex	
Female	127 (55.7)
Male	101 (44.3)
Marital status	
Single	129 (56.6)
Married	97 (42.5)
Divorced/ widow	2 (0.8)
Occupation	
University student	123 (53.9)
Employed	67 (29.4)
Unemployed	38 (16.7)
Hometown	
Hofuf	82 (36.0)
Al Mubarraz	73 (32.0)
Ahsa villages	73 (32.0)
Education level	
Bachelor's degree	144 (63.2)
Diploma	44 (19.3)
High school or equivalent	36 (15.8)
Less than high school	4 (1.8)
Household income	
Less than SR 6,000	72 (31.6)
SR 6,000-9,999	56 (24.6)
SR 10,000-13,999	56 (24.6)
SR 14,000-19,000	27 (11.8)
Over SR 19,000	17 (7.5)

Table 2 presents the responses of Saudi young adult participants regarding using social media networks as a source of nutrition information. Over 65% (n= 155) of the study participants were interested in increasing their nutrition awareness. Over 90% (n= 215) indicated that social media networks (Twitter, Instagram, and Snapchat) were the more beneficial tools for increasing nutrition knowledge than television, newspaper/magazine, or radio. Approximately 76% (n=173) of the study participants reported spending at least 1-3 hrs/day on social media. Instagram was the most frequently selected social media network that participants (n= 124) relied on to increase their nutrition knowledge. About 78% (n= 177) of the participants followed at least one Instagram account providing nutrition information. Regarding obtaining nutrition information on social media, 49% (n= 112) of the participants preferred videos, and 29% (n= 66) preferred infographic images.

**Table 2:** Assessment of the use of social media networks as a source for nutrition information in Saudi young adult participants (n=228)

Items	N (%)
Interested in increasing nutrition awareness	
Agree	155 (68.0)
Sometimes	63 (27.6)
Disagree	10 (4.4)
Most beneficial tool to increase nutrition knowledge	
Social media networks (Twitter- Instagram- Snapchat)	215 (94.3)
Television	6 (2.6)
Newspapers & magazines	6 (2.6)
Radio	1 (0.4)
Daily time to surf social media	
1-3 hrs/day	77 (33.8)
4-6 hrs/day	96 (42.1)
7-9 hrs/day	34 (14.9)
Over 10 hrs/day	21 (9.2)
Most social media networks rely on obtaining nutrition information	
Instagram	124 (54.4)
Twitter	69 (30.3)
Snapchat	35 (15.4)
Prevalence of following Instagram accounts that provide nutrition information	
None	51 (22.4)
One account	36 (15.8)
2-4 accounts	76 (33.3)
Over 4 accounts	65 (28.5)
Preference for the format of nutrition information	
Video	112 (49.1)
Infographic images	66 (28.9)
Text	42 (18.4)
Podcast	8 (3.5)
Most popular nutrition topics interested in	
Healthy nutrition in general	152 (66.5)
Dieting and weight loss	49 (21.5)
Calorie calculation & ideal weight	17 (7.5)
Therapeutic nutrition of diseases related to nutrition (diabetes, high blood pressure)	9 (3.9)
News on courses and activities on nutrition	1 (0.4)
Overall effect of social media on health behaviors	
Positively	163 (71.5)
Negatively	6 (25.9)
No change	59 (2.6)
Social media implementation of health behaviors	
Improving dietary habits	142 (62.3)
Practicing physical activity regularly	40 (17.5)
Losing weight	36 (15.8)
Quitting smoking	6 (2.6)
No effect	4 (1.8)
Helping ways of social media accounts	
Acquiring new dietary behaviors	108 (47.4)
Clarifying dietary behaviors	99 (43.4)
No help	21 (9.2)
Effect of social media on dietary behaviors	
Concerning the quantity and quality of food meals	86 (37.7)
Drinking 8 glasses of water daily	51 (22.4)
Selecting smart food choices	45 (19.7)
Consuming fruits & vegetables daily	22 (9.6)
Reading nutrition labels	22 (9.6)
No effect	2 (0.9)
Challenges of using social media for nutrition information	
Unproven information with a lack of evidence	90 (39.5)
Misleading or inaccurate nutrition information	74 (32.5)
Nutrition information provided by non-professionals	63 (27.6)
None	1 (0.4)
Physical activity limited due to surfing social media	
Agree	50 (21.9)
Disagree	79 (34.9)
Sometimes	94 (41.2)
Not sure	5 (2.2)

The study participants were asked about the most popular nutrition topics they were interested in. About 67% (n= 152) of the participants were interested in topics related to healthy nutrition in general, and about 22% (n=49) of the participants were interested in dieting and weight loss. Over 70% (n= 163) of the participants perceived that social media positively affected their dietary behaviors. In particular, 62% (n= 142) of the participants indicated that social media affected their health by improving their dietary behaviors. However, about 18% (n= 40) of the participants indicated that social media affected their health by practicing physical activity regularly.

Over 90% (n= 207) of the study participants perceived that social media helped them acquire new dietary behaviors and clarify dietary behaviors. Specifically, most of the participants perceived that social media improved their dietary behaviors regarding the quantity and quality of food meals of the participants (37.7%; n= 86), drinking eight glasses of water daily (22.4%; n= 51), and selecting smart food choices (19.7%; n= 45).

Nevertheless, the common challenges in obtaining nutrition information on social media faced by the participant included unproven information with a lack of evidence (39.5%; n= 90), misleading or inaccurate nutrition information (32.5%; n= 74), and nutrition information provided by non-professionals (27.6%; n= 63). Most participants (63%; n=144) reported that their physical activity might be limited due to surfing social media networks.

The results of the censored Tobit model are shown in Table 3. The predicted BMI value of men was 3.22 points higher than women, and the predicted value of BMI score for a single woman was 9.26 points less than a divorced woman. The predicted BMI score for a participant whose educational level was high school was 7.10 points higher than a participant with a college degree. Participants who relied on social media to get nutrition information had a predicted BMI score of fewer than 4.30 points compared to participants who depended on newspapers and magazines to obtain nutrition information.

**Table 3:** The association between socio-economics and social media variables and participants’ body mass index (BMI) *** indicates p<.01, and ** indicates p<.05

Variable	Tobit coefficient (beta)
Intercept	31.909*** (8.846)
Age	0.0761 (0.286)
Sex: male	3.222*** (0.735)
Marital status: married	-7.414 (5.272)
Marital status: single	-9.265 (5.237)
Marital status: widow	1.489 (7.823)
Education level: less than high school	7.095** (3.079)
Education level: high school	1.026 (0.965)
Education level: Diploma	0.424 (0.895)
Income: over SR 19,000	0.176 (1.471)
Income: SR 10,000-13,999	-0.268 (0.933)
Income: SR 14,000-18,999	-0.826 (1.179)
Income: SR 6,000-9,999	1.431 (0.904)
Most beneficial tool to increase nutrition knowledge: radio	0.177 (5.480)
Most beneficial tool to increase nutrition knowledge: social media	-4.302** (2.106)
Most beneficial tool to increase nutrition knowledge: TV	-5.475 (3.036)
Daily time to surf social media: 4-6 hrs/day	1.749** (0.787)
Daily time to surf social media: 7-9 hrs/day	2.261** (1.074)
Daily time to surf social media: over 10 hrs/day	0.587 (1.284)
Preference forms of nutrition information: podcast	0.228 (1.944)
Preference forms of nutrition information: text	1.148 (1.004)
Preference forms of nutrition information: video	1.981** (0.794)

Furthermore, participants who received nutrition information from TV had a predicated BMI score of 5.47 points less than those who relied on newspapers and magazines to obtain nutrition information. Participants who surfed social media at least 4 hours a day had higher predicted BMI scores than those who surfed social media for 1-3 hours a day. Lastly, participants who preferred the contents of nutrition information to be in videos had a predicted BMI score that was 1.98 points higher than those who preferred infographic images.

## Discussion

Social media networks have become a significant component of internet use by young adults, who are socially exposed to different information, including health, wellness, and nutrition [6,16]. Other research in Australia, the United States, Canada, and Norway indicated that most internet users searched for dietary information [17-21]. The current study assessed the use of social media networks as a source of nutrition information in 228 Saudi young adults in Al-Ahssa Province and examined the association between socio-economics and social media variables and participants’ BMI.

Most of the study participants attempted to increase their nutrition awareness using social media networks (Twitter, Instagram, and Snapchat) and found them beneficial tools, especially Instagram, for nutritional information. In research with other populations, Facebook and Twitter were identified as preferred social media networks for young adults and showed remarkable outcomes in enhancing audience engagement [1,2,19]. Even though the current study and other studies have shown that young adults would likely be receptive to using these platforms for nutrition information and education, there is a need for health professionals and nutrition educators to develop messages that take advantage of this delivery method for this age group.

It has been suggested that young adults are interested in using social media for learning about nutrition-related information, new recipes, and healthy eating [2]. The most frequently cited topics the study participants were interested in included healthy nutrition in general, dieting and weight loss, acquiring new dietary behaviors, and clarifying current dietary behaviors. Topics such as healthy food, calorie counting, and workouts have also been reported by Pappa et al. for Brazilian social media users [22]. Scanning and sharing information related to diet plans and exercise routines to stay fit on Facebook and Twitter were also reported by Zhang in university students [16]. Nutrition education can support these interests by providing young adults with the foundational knowledge necessary to make positive choices regarding their interests, mainly provided in videos and infographics. In addition, since young adults would be able to see the healthy dietary behaviors of others on social media networks, they would assume their performance of following healthy dietary behaviors would similarly be exposed to others. As the engagement with the online nutritional educational platform increases, they would gain more knowledge in the process [6]. In general, young adults are receptive to information about improving poor eating habits and body image or weight [23].

Young adults participating in the current study felt bombarded and challenged by misleading and unproven nutritional information with a lack of supporting evidence and misleading nutrition information provided by non-professionals. Such unqualified social media influencers could spread unsubstantiated claims about nutrition to a vulnerable audience. However, social media networks can act as a platform for nutrition educators to circulate trustworthy and credible nutrition information, interventions, and health promotion campaigns and encourage young adults to participate and engage with interventions [2]. It is necessary for nutrition educators and nutritionists to work with communications, media, and marketing professionals to understand how to interact and engage with young adults to alter and improve their dietary and activity behaviors.

The results of the censored Tobit model used in the current study showed that several socio-economic and social media variables were associated with the study participants' BMI. Single women were likelier to have lower BMI than men and divorced women. In addition, the study participants with a college degree were likelier to have lower BMI than those whose educational level was high school. Participants who relied on social media networks to obtain nutritional information and surfed social media networks for only 1-3 hours a day were likelier to have lower BMI than those who relied on newspapers and magazines and those who surfed social media networks for at least 4 hours a day. More time spent on social media might be related to sedentary lifestyles, especially since about 50% of participants agreed that their physical activity was sometimes limited due to the time spent using social media.

Furthermore, the study participants who preferred the contents of nutritional information to be in infographic images were likelier to have lower BMI than those who preferred the contents of nutritional information to be in videos. Generally, the internet has been identified as an important source of information and support for overweight and obese individuals [20]. The findings on social media use and BMI among young adults will set the stage for more research, given the popularity and effect of social media on nutrition.

Nutrition knowledge is essential, as it is the foundation for healthy dietary and lifestyle choices [24]. It had been previously reported that those using the internet for nutrition information were more likely than nonusers to be willing to learn ways to prepare healthy foods and choose healthier foods and lifestyles [25,20]. Nutrition education can be delivered through lectures, workshops, social campaigns, and technology, including social media [26-28]. During normal times, it may be difficult for participants to attend face-to-face classes on specific days and times due to work, school, and family obligations. During pandemic times, face-to-face classes may be prohibited or discouraged. Thus, social media is a relatively low-cost, direct, and flexible approach for nutrition educators to reach and motivate target populations [28,29]. The study participants were interested in nutrition education and used social media as a resource to meet their specific nutritional needs. It is necessary to keep up with trends in technology for young adults to ensure effective nutrition education and health promotion. Understanding how nutrition educators can take advantage of social media networks as an effective strategy to reach their target audience is needed, including best practices for implementation, management, and evaluation.

The study findings should be interpreted with some level of caution. The sample participants in this study were from Al-Ahssa Province, not representing other Saudi young adults from other provinces or countries. Convenience sampling was used in selecting the participants, limiting the level to which findings can be generalized. The BMI of the study participants was calculated based on self-reported height and weight, which may be inaccurate. In addition, the actual quality of the nutrition information received through social media networks was not assessed. More research (quantitative and qualitative) should be conducted with existing popular platforms to fully explore the potential of social media networks with Saudi young adults since the functionality of social media is more comprehensive and growing. Future research should also consider how to best engage with young adults using social media networks for making healthier dietary and lifestyle choices.

## Conclusions

Today, social media networks have been expanding and are widely accessed by young adults. This study explored the use of social media networks in Saudi young adults living in Al-Ahssa Province to obtain nutrition information. The study findings showed that this age group was keen to increase their nutrition awareness using social media networks (Twitter, Instagram, and Snapchat) and found them beneficial tools for different nutrition topics, such as acquiring new dietary behaviors and clarifying current dietary behaviors in the forms of videos and infographic images. However, young adults who participated in this study felt challenged by nutrition misinformation on social media provided by non-professionals. Nutrition experts, such as registered/licensed dietitians with specialized degrees in dietetics, nutrition, public health, or related sciences, would provide more accurate information related to nutrition and lifestyle topics.

In addition, several socio-economic variables (e.g., marital status and education levels) and social media variables (e.g., time spent on social media and preferred forms of nutritional information) were associated with the study participants' BMI. Given the popularity of social media and its flexible, no-contact delivery, nutrition educators have a potentially valuable tool for delivering effective interventions to support healthier choices that may contribute to improvements in BMI. Such interventions are especially needed at this critical life stage because of the new experiences many young adults face, such as preparing their meals. Thus, nutrition behaviors acquired at this age will significantly contribute to the quality of life in later years.
